# Direct analysis of lateral flow immunoassays for deoxynivalenol using electrospray ionization mass spectrometry

**DOI:** 10.1007/s00216-020-02890-4

**Published:** 2020-08-28

**Authors:** Ariadni Geballa-Koukoula, Arjen Gerssen, Michel W. F. Nielen

**Affiliations:** 1 Wageningen Food Safety Research, Wageningen University and Research, P.O. Box 230, 6700 AE Wageningen, The Netherlands; 2 Laboratory of Organic Chemistry, Wageningen University, Stippeneng 4, 6708 WE Wageningen, The Netherlands

**Keywords:** Lateral flow immunoassay, Mass spectrometry, Mycotoxins, Electrospray ionization, Confirmatory analysis, Biorecognition

## Abstract

Lateral flow immunoassays (LFIAs) are widely used for rapid food safety screening analysis. Thanks to simplified protocols and smartphone readouts, LFIAs are expected to be increasingly used on-site, even by non-experts. As a typical follow-up in EU regulatory settings, suspect samples are sent to laboratories for confirmatory analysis by liquid chromatography-tandem mass spectrometry (LC-MS/MS). However, re-analysis by LC-MS/MS is laborious and time-consuming. In this work, an identification LFIA (ID-LFIA) approach followed by quadrupole-orbitrap MS or triple quadrupole MS/MS analysis is presented. As a proof of concept, a dedicated ID-LFIA strip was developed for the mycotoxin deoxynivalenol (DON) following its initial screening by a commercial smartphone LFIA. The ID-LFIA strip can be simply immersed in the same sample extract used for the smartphone LFIA screening, and next, DON is retrieved from the monoclonal antibody with a dissociation solution consisting of methanol/ammonia. The solution thus obtained was analyzed directly in MS in order to rapidly confirm the presence of DON and any cross-reacting species. The protocol developed is capable of coping with severe ion suppression caused by LFIA buffers and nitrocellulose substrate residues. Initial analysis of blank, spiked, and incurred samples showed that the newly developed ID-LFIA-MS method was able to confirm the presence or absence of mycotoxins in the samples previously analyzed by LFIA and also differentiate between DON and DON 3-glucoside yielding the positive screening result. The concept and technique developed are envisaged to complement on-site screening and confirmation of any low molecular weight contaminant in future food control frameworks.

**Electronic supplementary material:**

The online version of this article (10.1007/s00216-020-02890-4) contains supplementary material, which is available to authorized users.

**Figure:**
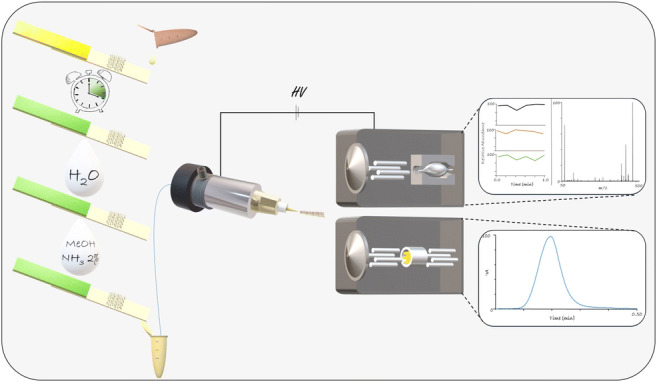
Graphical abstract

## Introduction

The EU General Food Law stipulates that in order to reassure safety within the food and feed chain, producers are responsible for conducting all necessary tests so that their products comply with the legislation. Also, regulatory authorities perform inspections to reassure compliance with the current regulations [1]. The present strategy for food contaminants monitoring often consists of a two-step approach. First, a rapid initial screening is performed, and second, confirmatory analysis of the suspect samples is carried out by instrumental analysis that provides unequivocal identification and quantification, if needed [2].

Many (bio)analytical techniques can be used as screening tools, such as biosensors that are based on specific biochemical recognitions from biomolecules such as antibodies, receptors, enzymes, or aptamers [3–5]. An example of a biosensor that employs antibodies is a lateral flow immunoassay (LFIA). LFIAs are widely used for on-site screening, with many applications reported, such as the detection of antibiotic residues, mycotoxins, or allergens [6–8]. Despite their extensive usage, a significant drawback of LFIAs is that the antibodies used can only recognize a specific part of the molecule, thus identifying groups of molecules, and making them unable to differentiate between molecules of similar physicochemical characteristics and structure [9–11]. This is the main reason why LFIAs are used solely for rapid screening, and if the result of the screening is ambiguous or suggests an exceeding of regulatory limits, then confirmation is necessary. The methods mostly used for confirmation are liquid or gas chromatography followed by tandem mass spectrometry (LC- or GC-MS/MS) [2, 12].

Screening LFIAs are relatively cheap, can be performed on-site, and are fast, and the readout of an LFIA can be performed visually or, as many developed lately, with the use of a smartphone camera, for a more reliable and semi-quantitative result [13]. Smartphones offer a variety of opportunities for user-friendly diagnostics, providing easy-to-read results, wireless data transfer to the cloud, and, most importantly for on-site testing, time and location data of the sampling [14]. Focusing solely on food safety, Rateni et al. recorded 27 studies, in only 5 years, between 2012 and 2017, that used smartphone-based diagnostics for food safety analysis [15]. However, the current food control strategy is limited by the elaborate, time-consuming confirmatory analysis follow-up, where tedious sample preparation, extraction, clean-up step, and chromatographic separation are needed [16–18]. Nonetheless, an LFIA itself can be considered an immunochromatographic device capable of selectively extracting analytes of interest, thus providing the necessary analyte selection and isolation step prior to instrumental analysis.

In this study, the concept of an identification LFIA (ID-LFIA) has been developed. With the ID-LFIA, the analytes are immuno-extracted and dissociated and subsequently, directly analyzed by MS without prior time-consuming chromatographic separation. The ID-LFIA can provide information on the identity of the bound analyte during the initial (smartphone) LFIA screening. Nevertheless, such a concept is far from trivial, due to the presence of involatile buffer salts and detergents in both the LFIA assay buffer and the nitrocellulose strip material that may hamper the MS analysis [19]. As a model system, the concept has been developed for the mycotoxin deoxynivalenol (DON), and consequently, the ID-LFIA contains monoclonal antibodies (mAbs) against DON. The model analyte DON is a mycotoxin produced by the *Fusarium* sp*.* fungi. Because of DON’s toxicity, maximum residue limits (MRLs) for DON in food and feed have been established to protect consumers [20]. Apart from DON, conjugated forms, such as DON 3-glucoside (DON3G), may occur in contaminated cereal crops, such as wheat, barley, and maize, as well as products thereof [21, 22].

Although a few approaches, using antibodies as a biorecognition element, and direct or ambient mass spectrometric identification, have been reported previously [23–26], to the best of our knowledge, this is the first successful attempt to simply analyze an LFIA with direct ESI-MS.

## Materials and methods

### Chemicals and reagents

Acetonitrile (ACN), methanol (MeOH), and water (H_2_O), all of UHPLC-MS purity grade, as well as hydrochloric acid (HCl), sodium hydroxide (NaOH), ammonia solution 25% (NH_3_), and formic acid 98% (HCOOH) were purchased from Merck (Darmstadt, Germany). Milli-Q water of 18.3 MΩ/cm conductivity was obtained using a water purification system from Merck (Amsterdam, The Netherlands). Bovine serum albumin (BSA) and bromothymol blue sodium salt solution in water were purchased from Sigma-Aldrich (Zwijndrecht, The Netherlands). A stock solution of 10× phosphate-buffered saline (PBS) containing 137 mM sodium chloride (NaCl), 2.7 mM potassium chloride (KCl), 10 mM sodium hydrogen phosphate (Na_2_HPO_4_), and 1.8 mM potassium dihydrogen phosphate (KH_2_PO_4_) (Merck, Darmstadt, Germany) and having a pH of 7.4 was prepared in Milli-Q water. Tenfold dilution of the stock solution to 1× PBS in Milli-Q water and addition of different percentages of Tween-20 (Sigma-Aldrich, Zwijndrecht, The Netherlands) provided different running buffer compositions for surface plasmon resonance (SPR) and LFIA experiments.

For SPR experiments, an amine coupling kit was obtained from GE Healthcare (Uppsala, Sweden), containing 1 M ethanolamine, 1-ethyl-3-(3-(dimethylamino)propyl)-carbodiimide hydrochloride (EDC), and N-hydroxysuccinimide (NHS).

A commercially available DON smartphone-based LFIA kit, RIDA QUICK DON, including DON extraction buffer, was obtained from r-Biopharm (Darmstadt, Germany). RosaFast DON screening tests for DON and its running buffer were purchased from Charm Sciences Inc. (Lawrence, UK).

Standard stock solutions of 100 μg/mL DON, 25 μg/mL ^13^C-DON, and 50 μg/mL DON3G, all in ACN, and wheat flour DON blank certified reference material (Joint Research Centre) were all purchased from LGC standards (Wesel, Germany). A contaminated beer sample was kindly provided by the Institute of Chemical Technology (Prague, CZ) and a naturally incurred wheat sample was purchased from Trilogy Analytical Laboratories (Arnhem, The Netherlands). Blank grounded and slurry grounded wheat and grounded barley samples, previously analyzed by confirmatory LC-MS/MS analysis, were provided in-house. Spiked samples were produced by spiking parts of the blank samples extracts, at 200 ng/mL with DON or DON3G. Two mouse monoclonal antibodies for DON, clone 2 and clone 4, as well as DON conjugate with BSA (DON-BSA), were purchased from Aokin AG (Berlin, Germany).

### ID-LFIA development

For the construction of the ID-LFIA strip, an XYZ3060 BioJet & AirJet instrument (Biodot Inc., Irvine, CA, USA) was used with a spraying speed of 1 μL/cm. The selected mAb diluted in 1× PBS was sprayed at the center of a nitrocellulose (NC) membrane (HiFlow Plus HF13502, Millipore, Carrigtwohill, Ireland). To capture a high quantity of DON, fifteen identical lines of the same antibody type and dilution were sprayed, thus forming a rectangle zone of mAb on the membrane. The NC membrane was then secured on plastic backing (G & L, San Jose, CA, USA). After drying of the strips at room temperature, an absorbent pad (Schleicher & Schuell, Dassel, Germany) of 3-cm length was incorporated, slightly overlapping at the end with the NC membrane. In contrast to regular LFIAs, the ID-LFIA lacks visible test and control lines. So, in order to assure that the running buffer moved correctly through the NC membrane, the indicator bromothymol blue was incorporated in the absorbent pad as follows: the absorbent pad was soaked in the indicator solution and air-dried overnight at room temperature. The indicator changes color from yellow (pH < 6) when dry to light green (pH > 7.4) when in contact with the running buffer, and keeps the light green color when the ID-LFIA is dry again after sampling. Finally, the strips were cut at a 5-mm width using a CM4000 BioDot Guillotine (Biodot Inc., Irvine, CA, USA). The final ID-LFIA strips were packed in aluminum pouches with silica desiccation packs, heat-sealed, and stored in a fridge at 4 °C until further use. For the development of the ID-LFIA, results from SPR experiments were evaluated and applied, which are provided in the Electronic Supplementary Material (ESM).

### Mass spectrometry

#### Quadrupole-orbitrap MS

Initial experiments for the optimization of ionization conditions were performed on a model Exactive orbitrap high resolution (HR) MS (Thermo Fisher Scientific, San Jose, CA, USA). The heated electrospray ionization (HESI) source parameters for the ionization of DON in negative ESI were optimized with direct infusion of a standard solution of DON 1 μg/mL in MeOH/NH_3_ (2%) at a constant flow rate of 20 μL/min. Then, the same optimized HESI source conditions were used for ionization of DON in the model Q-Exactive Focus quadrupole orbitrap HR-MS (Thermo Fisher Scientific). The following settings were used: sheath gas/aux gas 35/10 arbitrary units, spray voltage 2.5 kV, capillary temperature 270 °C, and capillary voltage − 50 V. Single ion monitoring (SIM) and MS-MS fragmentation (ddMS^2^) with a normalized collision energy of 10 for DON and 15 for the conjugated form DON3G were used as data acquisition methods. Spectra were recorded at a resolution of 70,000 FWHM at a 3 Hz scan rate with a maximum ion injection time of 1500 ms. The theoretical exact masses of the model analytes as well as the experimentally obtained *m/z* values for [M-H]^-^ precursor ions and fragment ions thereof are given in Table 1. Xcalibur software (Thermo Scientific) was used to obtain reconstructed ion chronograms (RIC) of the selected ions with 5 ppm mass accuracy, as well as the full scan mass spectra in the *m/z* range of 100–600 Da.

**Table 1:** Ions monitored for DON and its conjugated forms in quadrupole-orbitrap MS and the ion transitions in triple quadrupole MS

Analyte	Theoretical exact mass	Ion (negative ion mode)	Elemental composition	Quadrupole-orbitrap (*m/z*)	Triple quadrupole (*m/z*)
DON	296.1260	[M-H]^-^	[C_15_H_20_O_6_-H]^-^	295.1187	
		Fragment 1	[C_14_H_18_O_5_-H]^-^	265.1081	295.1 > 265.1
		Fragment 2	[C_14_H_16_O_4_-H]^-^		295.1 > 247.1
^13^C-DON	311.1763	[M-H]^-^	[C_15_H_20_O_6_-H]^-^	310.1690	
		Fragment 1	[C_14_H_18_O_5_-H]^-^	279.1551	310.2 > 279.2
DON3G	458.1788	[M-H]^-^	[C_21_H_30_O_11_-H]^-^	457.1715	
		Fragment 1	[C_20_H_28_O_10_-H]^-^	427.1610	457.0 > 427.0
		Fragment 2	[C_14_H_16_O_4_-H]^-^		457.0 > 247.1

#### Triple quadrupole MS/MS

The conditions of the ESI source were optimized on a Xevo TQ-XS Tandem Triple Quadrupole (QqQ) MS system (Waters Corporation, Milford, MA, USA) in full scan mode (*m/z* 100–600) using direct infusion of 1 μg/mL DON in MeOH/NH_3_ (2% v/v) with a constant flow rate of 20 μL/min. Fragmentation conditions were optimized in product ion scan mode using 100 ng/mL solutions in MeOH/NH_3_ (2% v/v) of DON and DON3G. Optimized conditions were as follows: capillary voltage 2.5 kV, cone voltage 5 V, source temperature 120 °C, desolvation temperature 200 °C, cone gas N_2_ flow 150 L/h, desolvation gas N_2_ flow 300 L/h, collision gas Ar flow 0.16 mL/min. Data were acquired in multiple reaction monitoring (MRM) mode with a collision energy of 11 eV for DON and 15 eV for DON3G. Final sample analysis was performed using flow injection analysis (FIA) with a 10-μL loop and MeOH/NH_3_ (2%) as mobile phase at a flow rate of 80 μL/min; total runtime was only 0.6 min. For data acquisition and processing of the MS data, MassLynx software (Waters) was used.

### Sample preparation

For wheat and barley samples, the extraction protocol from the r-Biopharm smartphone-based LFIA was used: 1 g of grounded wheat sample was extracted using 15 mL of extraction buffer from the assay kit. Slight agitation is needed followed by centrifugation in order to facilitate the sedimentation of the sample. For the extraction of the contaminated beer sample a method previously developed by Pagkali et al. was used [27], since the r-Biopharm protocol was not developed for the analysis of liquid samples. The degassed beer sample was simply diluted 8 times with the r-Biopharm extraction. In the r-biopharm LFIA, the extraction buffer doubles as a running buffer so directly following extraction, 100 μL is pipetted onto the sample port of the striptest. After 5 min, the result can be read visually and by using the smartphone app (r-Biopharm), the latter providing a quantitative result.

The ID-LFIA was further analyzed by MS. For the development of the ID-LFIA, 200 μL of the same sample extract is used for immersing the ID-LFIA strip in the sample, without any additional sample preparation needed. Next, the rectangle zone of mAb on the ID-LFIA was cut from the strip and placed in an Eppendorf tube with 500 μL of Milli-Q and slight agitation, to remove non-specifically bound analytes and minimize the ion suppression effects of assay buffer residues. Afterwards, the rectangle zone of mAb on the ID-LFIA was placed in an Eppendorf tube filled with 200 μL dissociation solution of MeOH/NH_3_ (2% v/v). After vortexing for 5 min and the addition of 40 ng/mL ^13^C-DON internal standard, to compensate for the ion suppression, the final solution is ready for analysis by direct ESI-MS.

To demonstrate the efficiency of this protocol in coping with assay buffer-induced ion suppression, the extraction protocol from a second LFIA provider, Charm Sc., was used in which 10 g of ground wheat sample was extracted with 50 mL of Milli-Q water, shaken for 1 min, and centrifuged. One hundred microliters of the extract was mixed with 1 mL of assay buffer, yielding the final solution used to develop the ID-LFIA according to the protocol described above.

## Results

### General concept

**Fig. 1:**
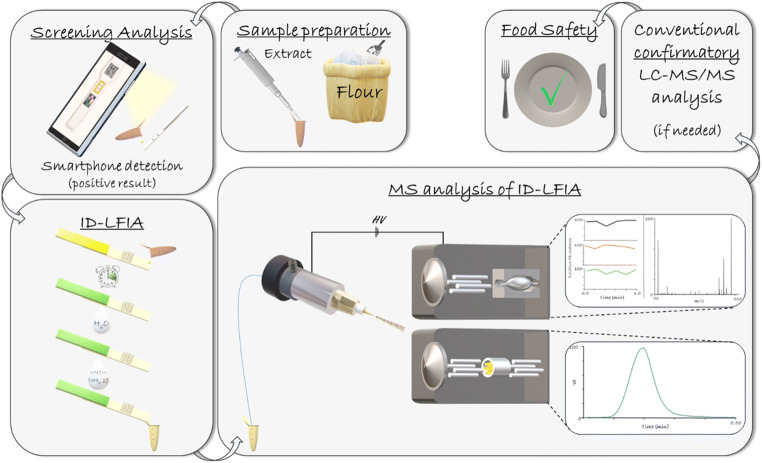
General concept of the ID-LFIA direct MS approach. After a simplified extraction of the sample, a regular or smartphone-based screening LFIA is performed. If the result is suspect (“positive”), then the same extract is used to develop the ID-LFIA. In the lab followed by washing, dissociation, and rapid direct analysis by Q-orbitrap MS, or QqQ-MS/MS. Only if more specification and/or a more accurate quantification is needed, then conventional time-consuming LC-MS/MS analysis is performed, for example, for the purpose of specific regulatory requirements

Direct coupling of a (smartphone-based) screening LFIA and MS is not straightforward. For low molecular weight analytes, such as the model analyte DON, the most common LFIA format is an indirect assay. In those assays, the mAb present in the conjugate pad competes with the analyte of interest in the sample and an immobilized analyte-protein conjugate on the test line. This assay format does not allow direct ionization of the analyte of interest from the test line since only the mAb is captured there [28]. Therefore, we decided to develop a complementary ID-LFIA, with anti-DON mAb, immobilized directly on the strip membrane for the subsequent detection and identification of DON by direct MS analysis. In this concept, the end-user may perform on-site a regular or smartphone-based LFIA and, in the case of a suspect result, immediately immerse our newly developed ID-LFIA into the same sample extract for identification in the lab later on (Fig. 1). The main benefit of this concept is the very rapid confirmation of the identity of the analyte(s) causing the suspect LFIA screening result. No laborious conventional LC-MS/MS is needed to check for false positive LFIA screening results. When, in the future, LFIAs are increasingly used on-site by non-experts, it is very important to overcome increased confirmatory analysis time and costs spent in the lab on increasing false positive screening samples.

### Direct electrospray MS of dissociated DON

For the mass spectrometric experiments, we first optimized the ionization conditions for DON using different standard solutions. Secondly, the compatibility of the dissociating solution of the ID-LFIA with ESI-MS and the matrix effects from both the LFIA assay buffers and the ID-LFIA strip material residues were investigated in detail.

Disruption of antibody binding can be, among others, achieved under acidic or alkaline conditions. Therefore, for evaluating the MS sensitivity of DON, we tested both 1 μg/mL DON in solutions of HCOOH (0.1% v/v), NH_3_ (0.1% v/v), and ammonium acetate/acetic acid buffer, and in different solvents such as MeOH and ACN, as well as mixtures of the organic solvents with H_2_O in 50/50 (v/v). The solution that yielded the highest MS intensity at optimized ion source conditions is MeOH/NH_3_ for the deprotonated ion of DON in negative ESI mode (ESM Fig. S3). Compared with the most intense ion in positive ESI mode, there was a 50-fold or higher increase in signal. Moreover, the final negative ESI conditions of 2.5 kV spray and − 50 V capillary voltages showed to be very robust, as only minor differences were observed for the intensity of the deprotonated ion of DON at the various capillary and spray voltages tested.

Next, we tested different percentages of NH_3_, and 2% v/v of NH_3_ in methanol was sufficient for optimal ionization, without altering the appearance of the spectra. Quantitative disruption of the immunocomplex by 2% v/v of NH_3_ in methanol was confirmed by SPR measurements (ESM Fig. S3), indicating that it should be feasible to dissociate DON from the mAb in the ID-LFIA for final MS analysis.

### Ion suppression caused by LFIA buffer and nitrocellulose substrate

**Fig. 2:**
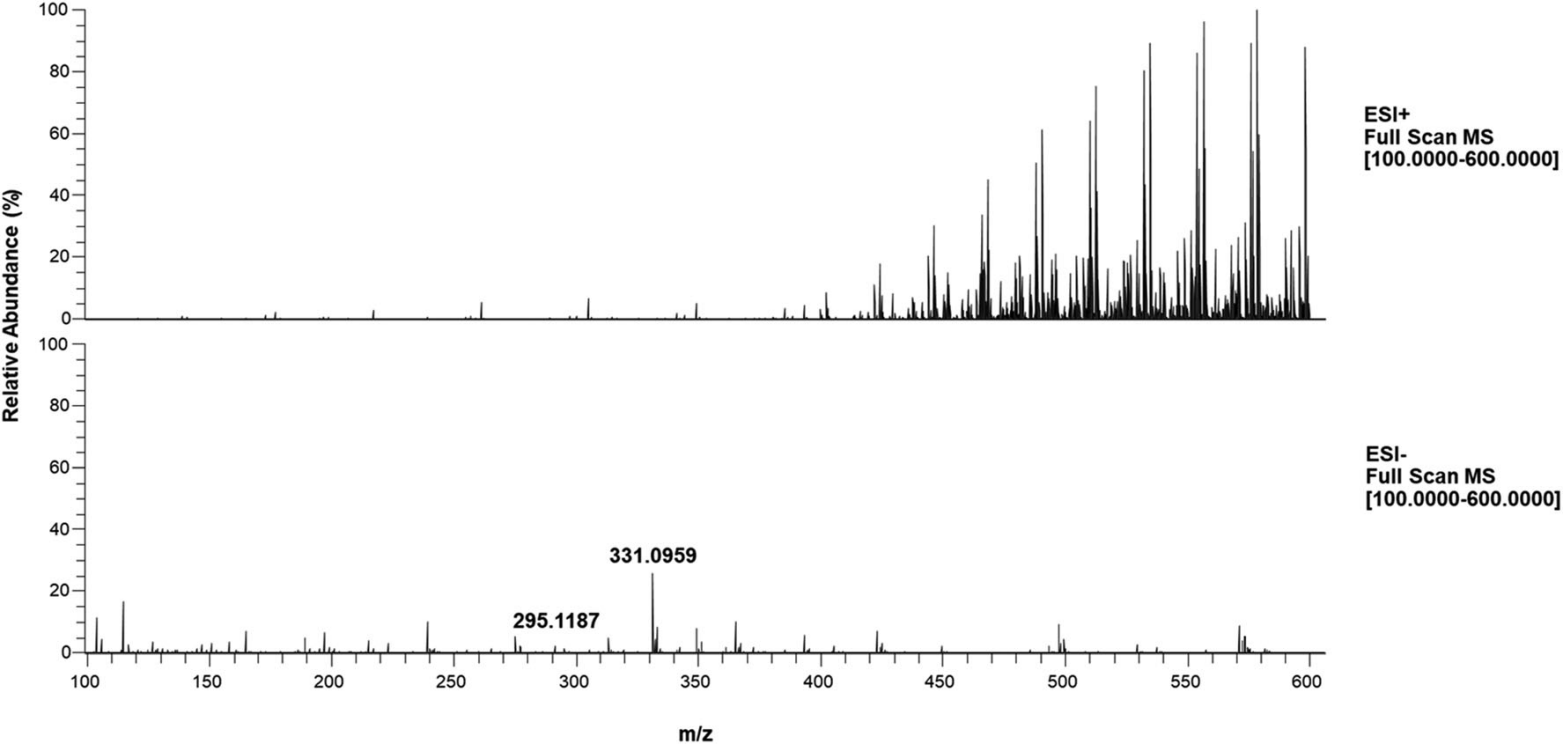
Full scan mass spectra (*m*/*z* 100–600), normalized on the highest intensity, of DON 1 μg/mL in MeOH/NH_3_ (2% v/v) solution containing 10% v/v r-Biopharm running buffer in positive ESI mode (above) and negative ESI mode (below)

Different types and percentages of LFIA running buffers, commonly used in screening assays, such as 1× PBS, were tested to assess the effect of residual buffer salts and detergents on the MS signal. Buffers used in immunoassays typically contain various non-volatile salts, such as sodium chloride or potassium phosphate [29], known to cause severe ion suppression in ESI-MS [19]. As expected, a higher percentage of buffers showed increased ion suppression, as well as increased background, regardless of the type of the buffer used (Fig. 2 and ESM Fig. S4). As can be seen in Fig. 2 (and ESM Fig. S5), the background caused by the surfactant is significantly higher in positive ion mode compared with the negative ion mode. In the negative ion spectra, both the [M-H]^-^ ion at *m/z* 295.1187 and the [M+Cl]^-^ ion of DON at *m/z* 331.0954 can be clearly observed, despite the ion suppression caused by the assay buffer. Aiming for a robust rapid analysis protocol, a washing step using 0.5 mL of Milli-Q water was incorporated to remove the excess of LFIA buffer components prior to dissociation with methanol/ammonia.

However, ion suppression may be caused not only by the LFIA buffer but also by residues from the nitrocellulose substrate. During the production of nitrocellulose membranes for LFIA use, proprietary additives are being used by manufacturers. And since nitrocellulose dissolves partly in methanol-ammonia during the dissociation step, membrane-embedded additives that cannot be entirely removed in the aqueous washing step may end up in the final solution for MS analysis. To evaluate the overall ion suppression caused by assay buffer and the nitrocellulose residues, we conducted a matrix-matched ion suppression study by comparing the intensity of the [M-H]^-^ ion of 40 ng/mL DON spiked in MeOH/NH_3_ (2% v/v) (reference solution) versus DON in a solution of an MeOH/NH_3_ (2% v/v) extract from a blank ID-LFIA strip developed with the assay buffer according to the ID-LFIA protocol. The results of the comparison showed an 80% drop in the DON’s intensity in the extract from blank ID-LFIA. In order to achieve still adequate sensitivity for the identification of DON in the less sensitive orbitrap MS, the ratio of captured DON molecules to buffer/strip material background was successfully managed by increasing the number of mAb lines on the ID-LFIA to 15, thereby creating a rectangular affinity trapping zone. By cutting that mAb zone from the strip prior to the dissociation with methanol/ammonia, we minimized the interference caused by dissolved nitrocellulose residues. As a result, in the final ID-LFIA-MS protocol and despite the lack of additional clean-up steps and chromatography, the sensitivity of DON to ^13^C-DON ratio dropped only by a factor of 2.5 and the linear regression only from 0.999 to 0.994 in the concentration range of 8–40 ng/mL. Remember that the ^13^C-DON is added just prior to the MS analysis and will compensate, at least partly, for ionization interferences but not for incomplete recovery from the immunocapturing and dissociation steps. The final sensitivity for DON thus obtained in ID-LFIA-MS is adequate for identification at regulatory limits.

Assuming 100% recovery from the mAb, an absolute quantity of 0.28 ng of DON is captured in a single line of mAbs. The number of lines increased the absolute quantity of analytes trapped by the mAb and the concentration of analytes in the sample solution for MS analysis. Calculations were made based on (i) the sensitivity of the orbitrap MS taking into account the ion suppression conditions, (ii) the maximum mAb loading and trapping capacity per line, and (iii) the required regulatory limits. Based on these calculations, an immunocapturing area composed of a number of 15 lines was found to be fit-for-purpose. In the final protocol, 200 μL MeOH solution containing 2% v/v NH_3_ solution was used for the dissociation and recovery of DON from the mAb on the strip. The calculated theoretical concentration of DON, assuming an extracted sample containing 1750 μg/kg DON and 100% recovery of DON from the 15 lines of mAb following dissociation from the mAb and aqueous washing, will be approximately 100 ng/mL in the absence ion suppression. In combination with the expected ion suppression from the substrate, we expect a signal beyond the LOD and LOQ (13 and 38 ng/mL, respectively) of the orbitrap MS. This will allow MS identification of DON and/or its conjugates in samples previously screened suspect by smartphone-based LFIA but not a precise and accurate quantitation.

### Direct Q-orbitrap HRMS and QqQ-MS/MS analysis of real samples from ID-LFIA

In the final quadrupole-orbitrap HRMS experiments, we chose to acquire deprotonated ions [M-H]^-^ in single ion monitoring (SIM) mode, followed by MS-MS measurement of the characteristic fragment ion of each analyte (Table 1). Apart from a hybrid quadrupole-orbitrap MS, we also used a triple quadrupole (QqQ) MS/MS, being the most frequently used MS technique in confirmatory food analysis [30–33]. For negative ESI-MS/MS detection, the MRM data acquisition mode was used, at the optimized conditions given in the “Material and methods” section.

Following the developed approach, both naturally contaminated and spiked samples were analyzed. In Q-orbitrap MS, a blank certified reference wheat material extract was spiked at 200 ng/mL for DON3G and 100 ng/mL for DON. The sample was analyzed 6 times to demonstrate the repeatability of the ID-LFIA-Q-orbitrap procedure. For the triple quadrupole measurements, 6 different blank wheat samples were analyzed, as well as spiked versions thereof, at 200 ng/mL DON. Moreover, in order to demonstrate that the developed ID-LFIA-MS protocol is independent of the running buffer composition and applicable to different sample matrices, we also analyzed ID-LFIAs developed with 1× PBST (0.05% v/v Tween-20), HEPES, the running buffer from the commercial Charm DON assay, and ID-LFIAs developed with barley extracts. Finally, naturally contaminated wheat and beer samples were analyzed in both instruments.

**Fig. 3:**
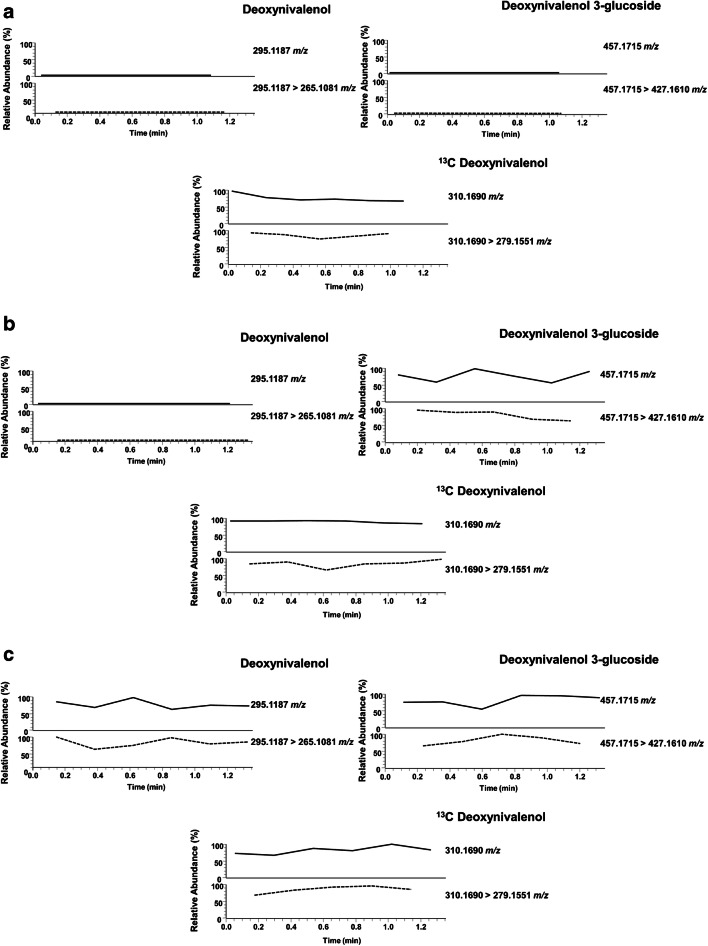
ID-LFIA-HRMS reconstructed ion currents for DON and DON3G, and fragment ions thereof, in (**a**) blank wheat, (**b**) DON3G spiked wheat, and (**c**) an incurred beer sample. ^13^C-DON added as a quality control internal standard prior to MS analysis. The deprotonated ion is shown in continuous line and the main fragment ion in dashed line

**Fig. 4:**
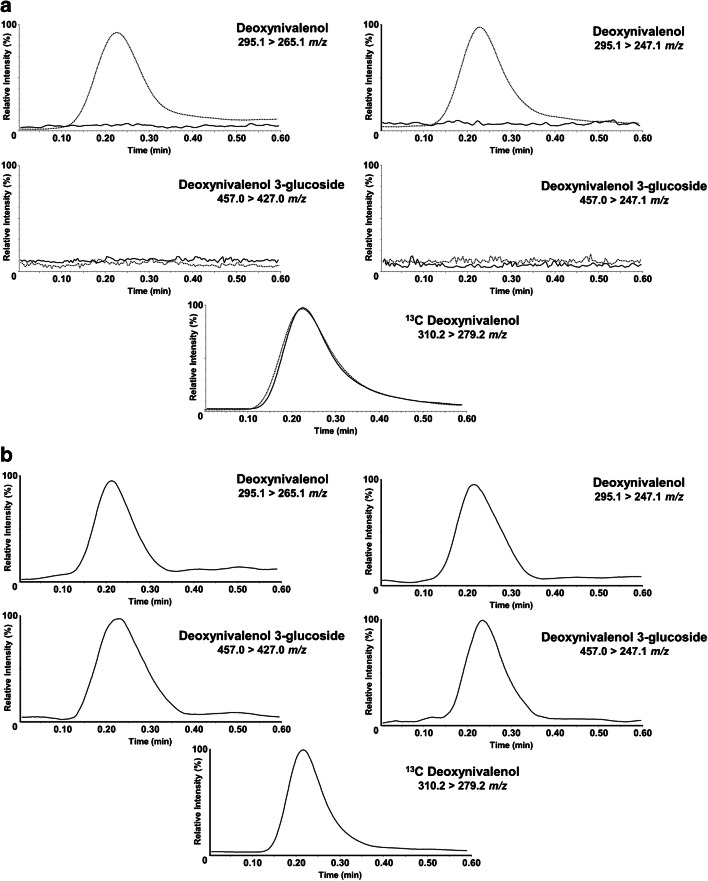
ID-LFIA-MS/MS reconstructed MRM transition ion currents of DON and DON3G in (**a**) spiked wheat (dashed line) overlayed with blank wheat (continuous line) and (**b**) incurred beer sample. ^13^C-DON added as a quality control internal standard prior to MS analysis

Using the extraction protocol described in the “Materials and methods” section, DON was isolated from the blank and spiked samples, and the extract was analyzed in duplicate by both the DON LFIA with smartphone readout and the newly developed ID-LFIA followed by direct MS analysis. Thanks to the smartphone camera and app, a semi-quantitative result is obtained, next to the visual readout, which can be compared with the MS analysis. The results are shown in Tables 2 and 3 and Figs. 3 and 4. In all cases, negative and suspect smartphone LFIA screening results were successfully confirmed by ID-LFIA-MS analysis: blank samples did not show any DON or DON conjugate in the MS analyses (Figs. 3a and 4a), while ID-LFIA-MS analysis of spiked (Figs. 3b and 4a) and incurred (Figs. 3c and 4b) samples showed characteristic deprotonated and fragment ions, allowing rapid confirmation of identity based on accurate mass (Table 2) and ion ratio data (Table 3). From Table 2, it can be seen that the repeatability of the DON signal for the six (identical) spiked wheat samples in ID-LFIA-HRMS was 7.2% RSD without correction for the ^13^C-DON internal standard. The repeatability of the ion ratio of *m/z* 295.1187 and 265.1081 in ID-LFIA-HRMS was 17% RSD. In ID-LFIA-QqQ-MS/MS, the robustness of the ion ratio turned out to be excellent for confirmation of identity according to regulatory requirements (Table 3). Moreover, neither the stability of the ion ratio nor the stability of the response factor versus the ^13^C-DON quality control standard was affected by the sample matrix or by the assay buffer composition (Table 3). The smartphone LFIA screening app reported a positive DON result of more than 5.5 mg/kg for the incurred beer sample. However, according to the ID-LFIA-MS follow-up analysis, this beer sample was found to contain apart from DON also DON3G (Fig. 3c and Fig. 4b), thereby underlining the added value of rapid MS identification of suspect LFIA screening assays. These results are in very good agreement with data from biochip spray MS and conventional confirmatory LC-MS/MS analysis, in which the same beer sample was found to contain 3.8 mg/mL of DON3G and 2.8 mg/mL of DON [25].

**Table 2:** Results from ID-LFIA-Q-orbitrap MS analysis of wheat and beer samples

Sample	Mean absolute intensity of peak height ± SD				LFIA screening result (mg/kg, mean ± SD)
	DON		DON3G		
	*m/z*				
	295.1187	265.1081	457.1715	427.1610	
Blank wheat	-	-	-	-	< 0.50 (± 0.00)
Spiked DON wheat (1)	1045 (± 5.0)	338 (± 8.5)	-	-	2.63 (± 0.09)
Spiked DON wheat (2)	1311 (± 29.0)	293 (± 5.5)	-	-	2.48 (± 0.01)
Spiked DON wheat (3)	1046 (± 64.0)	291 (± 17.5)	-	-	2.57 (± 0.13)
Spiked DON wheat (4)	1710 (± 250.0)	473 (± 22.5)	-	-	2.35 (± 0.00)
Spiked DON wheat (5)	1665 (± 130.0)	564 (± 3.5)	-	-	2.54 (± 0.04)
Spiked DON wheat (6)	1550 (± 135.0)	507 (± 41)	-	-	2.56 (± 0.11)
Spiked DON wheat—1-week stability of developed ID striptest	1580 (± 50)	994 (± 47)	-	-	2.60 (± 0.01)
Incurred wheat DON	1165 (± 45)	287 (± 21)	-	-	2.57 (± 0.00)
Spiked DON3G wheat	-	-	2230 (± 30)	983 (± 77)	2.64 (± 0.00)
Incurred beer UCT	3360 (± 220)	745 (± 34)	2900 (± 140)	820 (± 2)	> 5.50 (± 0.00)

Conditions: duplicate infusions of the same final ID-LFIA dissociation solution in Q-orbitrap MS. SD is the standard deviation of the duplicate measurement. The LFIA screening result was obtained with the r-Biopharm LFIA for DON and quantification with the associated smartphone application. SD is the standard deviation of duplicate reading of the same LFIA

**Table 3:** Results from ID-LFIA-QqQ-MS analysis of wheat, barley, and beer samples

Sample	Mean absolute intensity of peak area ± SD							Response factor (ratio of DON to DON ^13^C ± SD)	LFIA screening result (mg/kg, mean ± SD)
	DON			DON3G			DON ^13^C		
	*m/z*								
	295.1 > 265.1	295.1 > 247.1	Mean ratio of 265.1/247.1	457.0 > 427.0	457.0 > 247.1	Mean ratio of 427.1/247.1	310.2 > 279.2		
Spiked DON wheat (nr 798)	9364 (± 272)	2902 (± 17)	3.2 (± 0.07)	-	-	-	62,020 (± 2915)	0.16 (± 0.011)	3.95 (± 0.10)
Spiked DON wheat (nr 802)	9369 (± 674)	2880 (± 126)	3.2 (± 0.09)	-	-	-	62,162 (± 5931)	0.16 (± 0.004)	3.88 (± 0.00)
Spiked DON wheat (nr 803)	10,001 (± 135)	3056 (± 87)	3.3 (± 0.05)	-	-	-	60,964 (± 2053)	0.16 (± 0.003)	3.87 (± 0.03)
Spiked DON wheat (CRM)	7711 (± 280)	2348 (± 219)	3.3 (± 0.04)	-	-	-	48,680 (± 9654)	0.16 (± 0.005)	4.29 (± 0.17)
Spiked DON wheat (nr 607)	6600 (± 319)	1998 (± 96)	3.3 (± 0.00)	-	-	-	40,411 (± 1542)	0.16 (± 0.002)	3.88 (± 0.02)
Spiked DON wheat (nr 670)	4963 (± 90)	1532 (± 46)	3.2 (± 0.04)	-	-	-	31,230 (± 1397)	0.16 (± 0.004)	3.93 (± 0.19)
Spiked DON barley (nr 797)	4575 (± 72)	1420 (± 124)	3.2 (± 0.01)	-	-	-	34,073 (± 7429)	0.17 (± 0.021)	3.48 (± 0.10)
Spiked DON wheat (CRM)/1× PBST (0.05% Tween-20)	6816 (± 162)	2065 (± 100)	3.3 (± 0.08)	-	-	-	41,457 (± 1354)	0.16 (± 0.001)	-
Spiked DON wheat (CRM)/1× HEPES	6948 (± 142)	2026 (± 35)	3.4 (± 0.01)	-	-	-	41,199 (± 364)	0.17 (± 0.006)	-
Spiked DON wheat (CRM)/Charm Sc. r.b.	6154 (± 59)	1808 (± 164)	3.4 (± 0.00)	-	-	-	38,765 (± 1267)	0.16 (± 0.009)	-
Incurred wheat DON	5775 (± 250)	1748 (± 59)	3.3 (± 0.03)	-	-	-	34,873 (± 230)	0.17 (± 0.008)	3.56 (± 0.18)
Incurred beer UCT	3504 (± 219)	1078 (± 57)	3.3 (± 0.05)	410 (± 6.2)	400 (± 12.3)	1.0 (± 0.025)	21,139 (± 801)	0.16 (± 0.005)	> 5.5 (± 0.00)

Conditions: duplicate injections of 10 μL of the same final ID-LFIA dissociation solution were done in QqQ-MS with a flow rate of 80 μL/min. SD is the standard deviation of the duplicate measurements. The screening result was performed with the r-Biopharm LFIA and the quantification was done with the associated smartphone app. SD is the standard deviation of duplicate reading of the same LFIA

In confirmatory techniques, three identification points (IPs) are required for the so-called group B substances that are earned by measuring specific characteristics, as described in the legislation [2]. In our ID-LFIA-MS approach, when measuring in hybrid Q-orbitrap MS, two ions, namely, the precursor ion and a product ion, are monitored, thus yielding 4 IPs. With the QqQ, two product ions are monitored in MRM mode yielding 3 IPs. Even without chromatographic separation, the IP system can be applied to confirm the identity of the analyte causing the suspect LFIA screening result. Apart from that, additional IPs might possibly be granted in future legislation because of the inherent “immuno-chromatography” nature of the ID-LFIA.

## Discussion

A simplified direct analysis of an LFIA with MS was developed allowing the rapid identification of low molecular weight analytes previously screened suspect by regular or smartphone-based LFIAs. Supported by SPR studies, selective capturing of the target analyte by a mAb on a newly developed ID-LFIA striptest was achieved, followed by identification of the immuno-captured analyte, as well as any (un)expected cross-reacting conjugates, using either Q-orbitrap HRMS or QqQ-MS/MS. The developed ID-LFIA-MS protocol was found to rapidly confirm the identity of the analytes based on accurate mass and/or robust ion ratios that were not affected by different sample matrices nor by different LFIA buffer compositions. The ion suppression caused was successfully managed, by introducing multiple lines of mAb and addition of a washing step. Nonetheless, future experimentation with different types of porous substrates to capture the mAb and provide capillary flow, different types of rapid screening assays and subsequent direct analysis of the analytes with MS could be tested to provide more alternatives direct analytical approaches.

In the world of increasing numbers of simplified and smartphone-based food safety screening diagnostics, higher numbers of screening data will become available and, as a consequence, the number of results requiring a follow-up by instrumental analysis will increase as well, leading to more and more time-consuming confirmation analysis needed. Even though conventional LC-MS/MS analysis has higher multiplexing and quantitative potentials [16, 22, 33], the developed ID-LFIA-MS approach may act as an intermediate between the screening assays and the conventional quantitative confirmatory analysis by chromatographic separation followed by mass spectrometry, in order to moderate the increasing number of analyses. Following this concept, an individual performs a commercially available smartphone-based screening assay with LFIA format. In the case of a suspect or ambiguous result, one would simply immerse our newly developed ID-LFIA in the same sample extract and send the strip to the lab by courier, mail, or otherwise, for further processing. Following the wash and dissociation steps, the LFIA can be analyzed by direct MS in the lab, requiring less than a minute to either verify or reject the LFIA screening result as a false positive. Only if the result of this intermediate analysis is positive and further information or accurate quantification is needed, then conventional confirmatory analysis with LC-MS/MS would be necessary to be performed.

## Electronic supplementary material

ESM 1 (PDF 772 kb)
