# Supplementary material for: Direct analysis of lateral flow immunoassays for deoxynivalenol using electrospray ionization mass spectrometry
Source: Anal Bioanal Chem. 2020 Aug 28;412(27):7547–58. doi: 10.1007/s00216-020-02890-4 (PMC7533258; doi:10.1007/s00216-020-02890-4)
Supplement: Supplementary file 1 — (PDF 772 kb) [file 216_2020_2890_MOESM1_ESM.pdf]

Analytical and Bioanalytical Chemistry

Electronic Supplementary Material

Direct analysis of lateral flow immunoassays for deoxynivalenol using electrospray ionization mass spectrometry

Ariadni Geballa-Koukoula, Arjen Gerssen, Michel W.F. Nielen

Table of Contents

1. SPR measurements....3

1.1 Biosensor Chip Preparation....3

1.2 Antibody Screening Assay....3

1.3 Competitive inhibition method and cross-reactivity testing....4

1.4 Analyte dissociation form the mAb....5

2. MS ion suppression data....6

3. References....8

1. SPR measurements

1.1 Biosensor Chip Preparation

In order to develop the ID-LFIA approach, appropriate biorecognition conditions were determined using Surface Plasmon Resonance (SPR). All SPR measurements were performed using a Biacore 3000 (GE Healthcare, Uppsala, Sweden) with carboxymethylated dextran-coated gold chip (CM5) (GE Healthcare, Uppsala, Sweden) having four flow channels. Prior to immobilization onto the SPR CM5 chip, DON-BSA was diluted to 50 µg/mL in 10 mM acetate buffer of various pH's, respectively 4.0, 4.5, 5.0 and 5.5, and tested using the pH scouting wizard in the Biacore 3000 control software [1]. The optimum immobilization level was achieved at pH 4.5, as indicated by the higher response units (RU) achieved at the binding of the DON-BSA with the surface. This finding is, as expected, close to pH 4.7, which is the isoelectric point of BSA [2].

After the pH screening, immobilization in flow channels was performed using a slightly modified method from the previously described by Joshi et al. [3]. First, immobilization at flow channels 1 and 2 of the CM5 chip was performed with DON-BSA and BSA, respectively, instead of DON-OVA and OVA. Instead of HBS-EP, PBS with 0.02% Tween-20 was used as running buffer at a flow rate of 5 µL/min, in order to simulate better the conditions of the LFIA development. The immobilization starts by first activating the carboxymethylated surface of the CM5 chip, with a 1:1 mixture of EDC/NHS for 7 minutes, followed by a 7-minute injection of DON-BSA 50 µg/mL or BSA 50 µg/mL, diluted in 10 mM acetate buffer pH 4.5, aiming at 5000 RU relative to the running buffer's baseline. Following the immobilization, the remaining free activated carboxymethylated groups of flow channels 1 and 2 were blocked with ethanolamine 1 M injection for 7 minutes. Finally, the chips were stored at 4-8 °C until use.

1.2 Antibody Screening Assay

The mAbs, clone 2 and 4, diluted in running buffer at a concentration of 20 µg/mL, were screened for their affinity towards immobilized DON-BSA using PBS with Tween-20 (0.02%) at a flow rate of 5 µL/min. Of each mAb, 35 µL were injected and the binding was monitored under constant flow at 5 µL/min. The final step was the regeneration of the SPR chip surface by injecting 25 mM NaOH at a flow rate of 100 µL/min. According to the RU values in the SPR sensorgrams obtained (Figure S1), we can conclude that mAb clone 2 is binding with higher affinity to DON-BSA immobilized on the surface than clone 4. We observed that clone 2, was not dissociating; in contrast, clone 4 dissociated rapidly from the DON-BSA chip, demonstrating a stronger binding of the former.

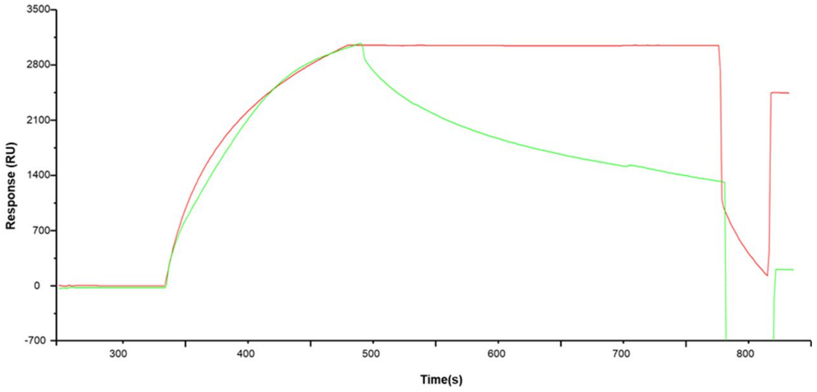

Fig. S1 SPR sensorgrams comparing the binding of mAb clones 2 (red) and 4 (green) to immobilized DON-BSA on the flow channel of the SPR chip

1.3 Competitive inhibition method and cross-reactivity testing

In order to assess the sensitivity of the selected antibody, a competitive inhibition method was implemented as described in reference [4]. A 35 µL mixture of mAb 2 µg/mL together with increasing concentrations of DON standard solutions diluted in running buffer (1:1 v/v) were injected in the flow channels 1 and 2. The concentration of DON ranged from 0.0001 µg/mL to 10 µg/mL, with steps of a 10-fold concentration increase. The experiment was performed in duplicate, with four blank solvent injections, two at the beginning and two at the end of the method. The results were processed with Biaevaluate (GE Healthcare, Uppsala, Sweden) and an inhibition curve was constructed using Prism (GraphPad Software Inc) (Figure S2).

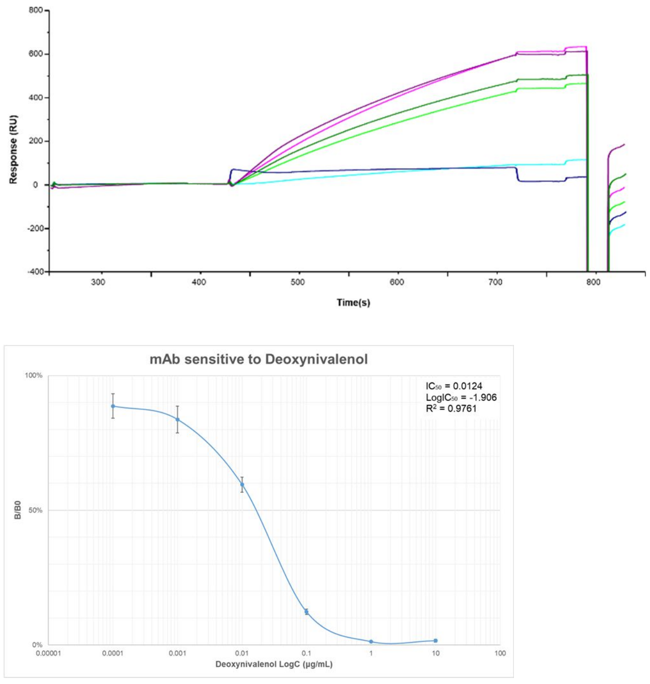

Fig. S1 Overlay of SPR sensorgrams obtained from the competitive inhibition assay for DON (top) and inhibition curve (bottom)

The cross-reactivity of the selected DON mAb with the available conjugated form of DON was also assessed. Briefly, 2 μg/mL solution of the mAb was mixed at 1:1 ratio with 0.1 μg/mL of DON3G. 35 μL of this mixture was injected into the flow channel at a flow rate of 5 μL/min. The conjugated form tested showed binding with the anti-DON mAb.

1.4 Analyte dissociation form the mAb

To assess the effective disruption of mAb-DON binding in the optimized solution of 2%v/v NH_3 in methanol, an SPR measurement was conducted. In the CM5 SPR chip, with immobilized DON-BSA, using PBS with Tween-20 (0.02%), as running buffer with a flow rate of 5 μL/min, 35 μL of clone 2 antiDON mAb was injected, followed by a 20 μL regeneration injection of MeOH/NH_3 2%. According to the RU values in the SPR sensorgrams obtained before and after the regeneration (Figure S3), we can conclude that the selected MeOH/NH_3 2% dissociation solution, is effective for a rapid and complete dissociation. The regeneration of the SPR chip requires approximately 3 minutes, so a total vortex time of 5 minutes and a 200 μL volume was chosen in the final ID-LFIA protocol to reassure complete dissociation of the analyte form the mAb.

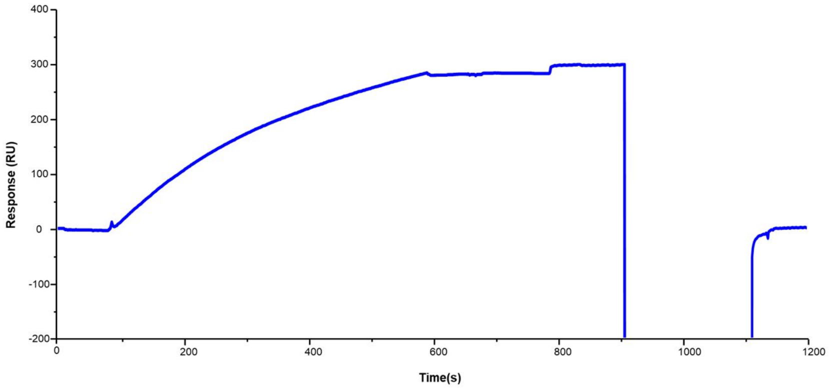

Fig. S3 SPR sensorgrams of the binding of mAb clones 2 to immobilized DON-BSA on the flow channel of the SPR chip, followed by regeneration with the dissociation solution MeOH/NH_3 2%. The RU value reaches the baseline after the regeneration

2. MS ion suppression data

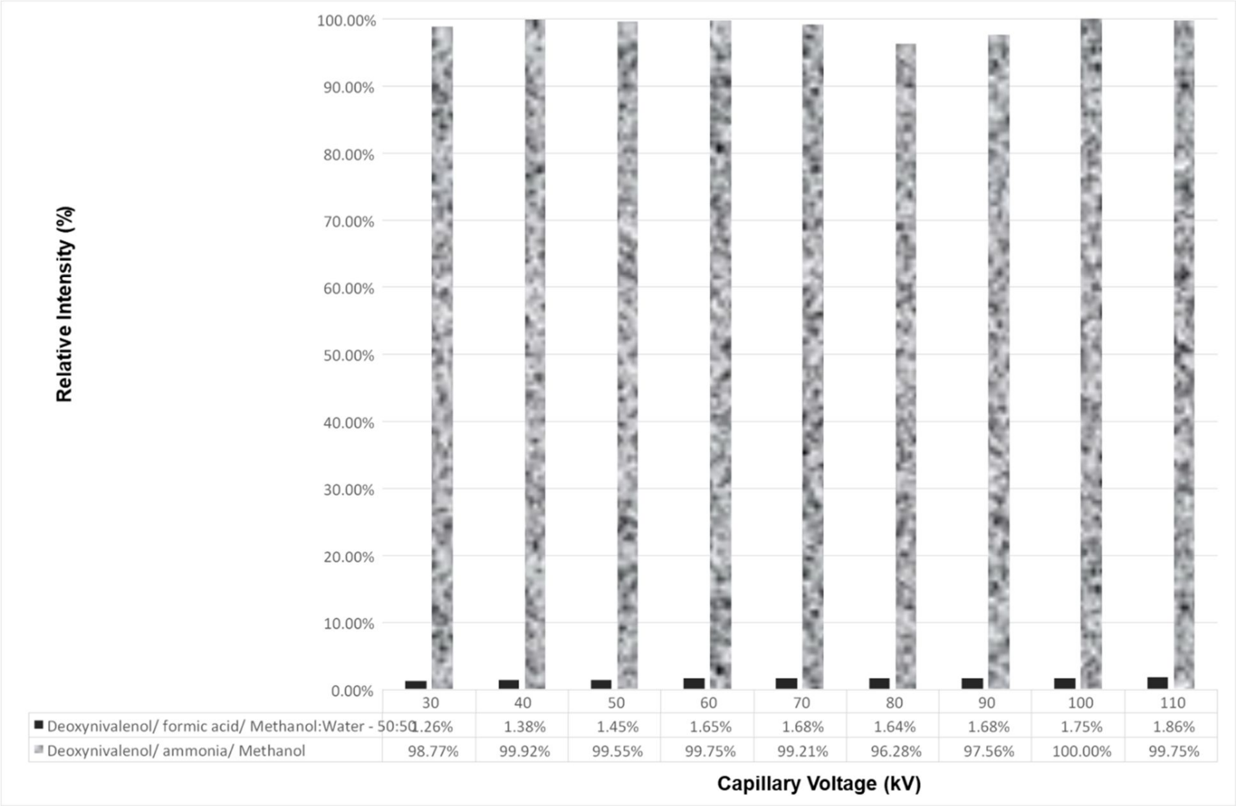

Fig. S4 Relative intensity of DON 1 µg/mL $[M-H]^-$ and $[M+H]^+$ in Methanol/ ammonia 0.1% v/v and methanol/ water/ formic acid 50/50/0.1 v/v/v, shown in grey and black bars, respectively. From all the different solutions of DON tested, those are the optimum for the ionization in negative and positive mode, recorded at 2.5 kV spray voltage, resolution of 50,000 FWHM and 4.5 kV spray voltage, resolution of 100,000 FWHM, respectively

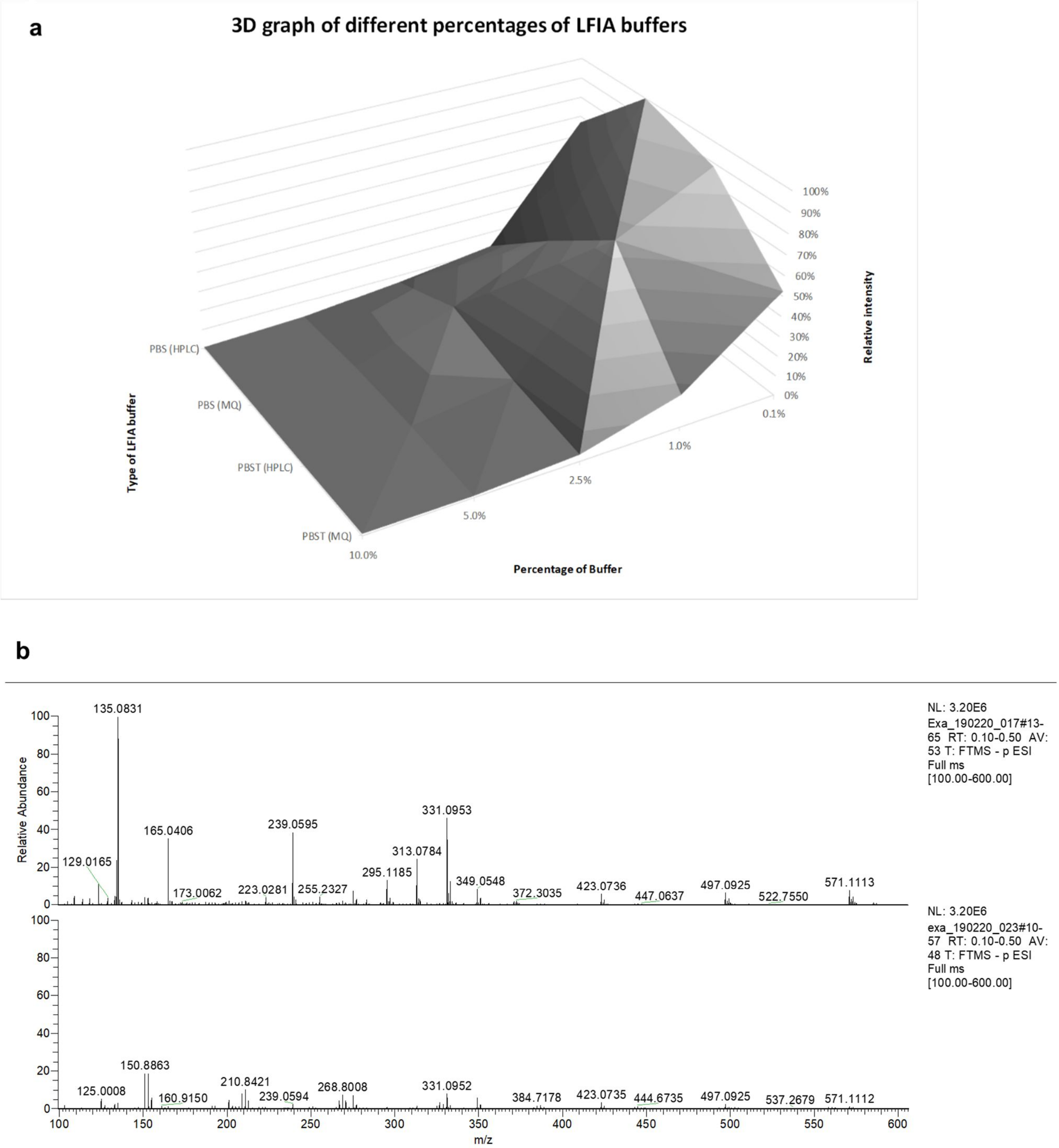

Fig. S5 (a) The relative intensity of [M-H]$^-$ of DON 1 µg/mL in standard solutions of MeOH/NH_3 2% containing different percentages of running buffer. The higher the percentage of the running buffer, the higher the ion suppression, regardless of the type and the purity of the buffer used. (b) Mass spectra of DON 1 µg/mL in MeOH/NH_3 2% containing 1% PBST (0.05% Tween-20) (above) and 10% PBST (0.05% Tween-20) (below). The spectra are normalized based on the higher intensity peak apparent in both spectra. The spectra with the higher percentage PBST yields lower signal because of the ion suppression. On the top spectra, we can see the [M-H]$^-$ of DON m/z 295.1187 as well as characteristic adducts [M+Cl]$^-$ m/z 331.0954 and [M+Cl]$^-$ isotope m/z 333.0924

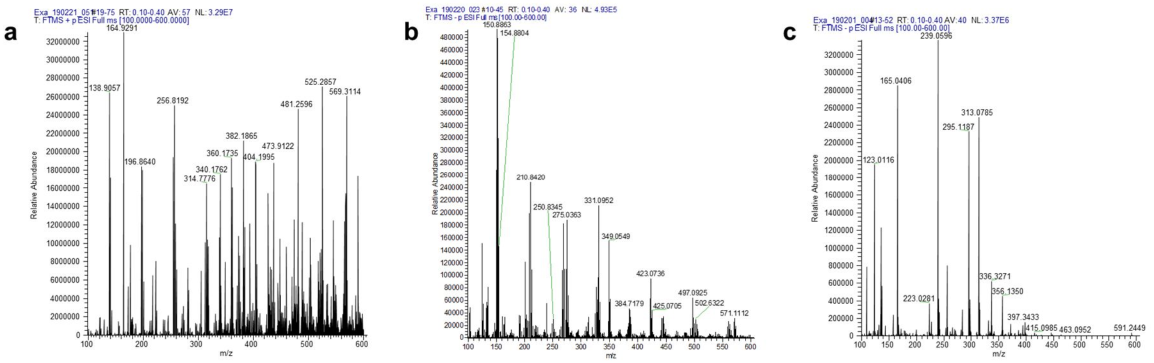

Fig. S6 Full scan mass spectra (m/z 100-600) of DON 1 µg/mL in MeOH/NH_3 2% solution containing 10% PBST (0.05% Tween-20) in (a) positive ESI mode (b) negative ESI mode and (c) DON 1 µg/mL in MeOH/NH_3 2%. The background in positive ionization mode is significantly higher compared to the spectrum in negative ion mode, and both spectra show surfactant ions. Nevertheless, in the negative ion spectra, the $[M\text{-}H]^-$ m/z 295.1187 and the $[M+Cl]^-$ m/z 331.0954 can be observed. The formation of the chlorine adduct can be attributed to the high concentration of the Cl in the sample because of the PBST buffer present

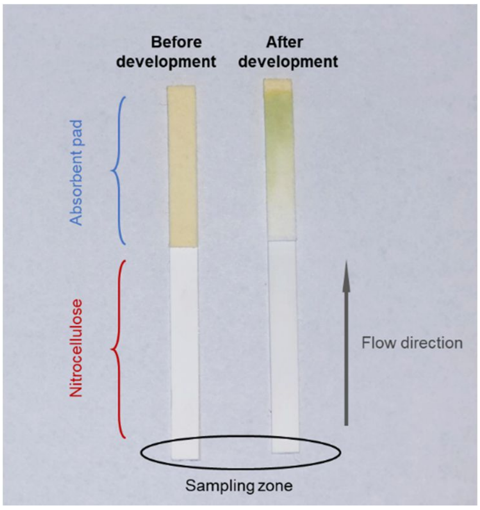

Fig. S7 The ID-LFIA strip in action. The light yellow absorbant pad (before the development of the stiptest), turning to light green (after development) when in contact with the running buffer

3. References

1. Tudos AJ, Lucas-van den Bos ER, Stigter EC (2003) Rapid surface plasmon resonance-based inhibition assay of deoxynivalenol. J Agric Food Chem 51 (20):5843-5848. doi:10.1021/jf030244d
2. Jahanban-Esfahlan A, Ostadrahimi A, Jahanban-Esfahlan R, Roufegarinejad L, Tabibiazar M, Amarowicz R (2019) Recent developments in the detection of bovine serum albumin. Int J Biol Macromol 138:602-617. doi:10.1016/j.ijbiomac.2019.07.096
3. Joshi S, Segarra-Fas A, Peters J, Zuilhof H, van Beek TA, Nielen MWF (2016) Multiplex surface plasmon resonance biosensing and its transferability towards imaging nanoplasmonics for detection of mycotoxins in barley. Analyst 141 (4):1307-1318. doi:10.1039/c5an02512e
4. Kadota T, Takezawa Y, Hirano S, Tajima O, Maragos CM, Nakajima T, Tanaka T, Kamata Y, Sugita-Konishi Y (2010) Rapid detection of nivalenol and deoxynivalenol in wheat using surface plasmon resonance immunoassay. Anal Chim Acta 673 (2):173-178. doi:10.1016/j.aca.2010.05.028
